# The molecular structural features controlling stickiness in cooked rice, a major palatability determinant

**DOI:** 10.1038/srep43713

**Published:** 2017-03-06

**Authors:** Hongyan Li, Melissa A. Fitzgerald, Sangeeta Prakash, Timothy M. Nicholson, Robert G. Gilbert

**Affiliations:** 1 Joint International Research Laboratory of Agriculture and Agri-Product Safety, College of Agriculture, Yangzhou University, Yangzhou 225009, Jiangsu Province, China; 2 The University of Queensland, Centre for Nutrition and Food Sciences, Queensland Alliance for Agriculture and Food Innovation, Brisbane 4072, QLD, Australia; 3 The University of Queensland, School of Agriculture and Food Science, Brisbane 4072, QLD, Australia; 4 The University of Queensland, School of Chemical Engineering, Brisbane 4072, QLD, Australia

## Abstract

The stickiness of cooked rice is important for eating quality and consumer acceptance. The first molecular understanding of stickiness is obtained from leaching and molecular structural characteristics during cooking. Starch is a highly branched glucose polymer. We find (i) the molecular size of leached amylopectin is 30 times smaller than that of native amylopectin while (ii) that of leached amylose is 5 times smaller than that of native amylose, (iii) the chain-length distribution (CLD: the number of monomer units in a chain on the branched polymer) of leached amylopectin is similar to native amylopectin while (iv) the CLD of leached amylose is much narrower than that of the native amylose, and (v) mainly amylopectin, not amylose, leaches out of the granule and rice kernel during cooking. Stickiness is found to increase with decreasing amylose content in the whole grain, and, in the leachate, with increasing total amount of amylopectin, the proportion of short amylopectin chains, and amylopectin molecular size. Molecular adhesion mechanisms are put forward to explain this result. This molecular structural mechanism provides a new tool for rice breeders to select cultivars with desirable palatability by quantifying the components and molecular structure of leached starch.

Rice is a major staple food world-wide. Consumer preferences are shifting towards better-quality rice, particularly towards varieties with good eating quality^1^. Rice texture is of prime importance to eating quality and consumer acceptance. Texture is a multi-faceted sensory property, with hardness and stickiness as the most commonly determined and discriminable textural properties of cooked rice^2^,^3^. Rice is the only major cereal that is most often consumed in the form of whole grain after cooking. In addition to sensory evaluation by human panels, textural properties of cooked rice are commonly evaluated by texture profile analysis (TPA) with a textural analyser^4^,^5^. TPA is a technique that has been extensively employed to mechanically and geometrically characterize food materials. The technique involves measuring the mechanical response during a double compression, which attempts to mimic the first and second bites of a food. For cooked rice, the two most meaningful parameters derived from TPA are hardness (the force required to attain a given deformation) and adhesiveness (a quantity that simulates the work required to overcome the attractive forces between the surface of the sample and the surface of the probe with which the same comes into contact)^6^.

Cooked rice texture is affected by a wide range of factors, such as the amylose content^7^, postharvest processing^8^ and cooking method^9^. For example, the method used to cook rice can vary between different regions, and is often specific to a varietal type^9^,^10^. South and East Asians always cook rice in a rice cooker with using a particular ratio of water (the absorption method); Indians prefer cooking rice by boiling it in excess water, and Americans like cooking rice in large amounts of water which is then drained. The absorption method with controlled volumes of water is applied in this study.

Starch structure has an important role in rice texture^4^,^5^,^11^,^12^. Starch, the main component of rice grains^5^, is a branched glucose polymer comprising two types of molecules: amylopectin (Ap) and amylose (Am). Ap molecules are highly branched with a vast number of short branches and relatively large molecular weights, ~10^7–8^, whereas Am has a smaller molecular weight (~10^5–6^) and a few long branches^13^. Starch biosynthesis is a complex pathway controlled by at least four different classes of enzymes: ADP-glucose pyrophosphorylase (AGPase), starch synthases (SSs), starch branching enzymes (SBE), and debranching enzyme (DBE). The biosynthesis of amylose is mostly controlled by granule-bound starch synthase (GBSS) while that of amylopectin is more complex, involving the combined actions of SS, SBE, and DBE^14^. Amylose content was, since the mid-1980s, considered to be the most important determinant of the hardness of cooked rice^7^. In the mid-1990s, it was proposed that hardness is more dependent on the long amylopectin chains^11^,^12^. Based on the significant role of amylose in determining the hardness of cooked rice, a set of different physicochemical methods has been developed to measure rice hardness, such as the starch-iodine blue value^15^, Brabender viscogram^16^, alkali spreading value^17^ and gel consistency^18^. In previous work, we found that the molecular fine structure of amylose, both the molecular size and chain-length distribution, are also significant determinants of the hardness of cooked rice^5^.

In contrast to hardness, stickiness between rice grains is less commonly investigated, and the mechanism of rice stickiness is unclear, even though stickiness between rice grains is the key requirement for sushi, which is a very popular food. Stickiness has previously been related to grain length, with short grains being usually thought of as sticky and the long grains as not^19^. Recent studies show that stickiness is always negatively correlated with amylose content and hardness, i.e. high-amylose rice is usually harder and less sticky while low-amylose rice is softer and sticky^2^,^3^,^4^,^5^. Nevertheless, rice cultivars with similar amylose contents can still display different stickiness^20^. Very few publications address the structural reasons for stickiness of cooked rice. Patindol *et al*.^2^ suggested that the amylose-amylopectin ratio of the leached materials during rice cooking may be the main indicator of cooked rice hardness and stickiness. Ayabe *et al*.^20^ compared the stickiness of two rice cultivars with similar amylose content, Nipponbare (Japonica rice) and Khao Dawk Mali (Indica rice), and suggested that the difference in the amount of leached materials from the surface of cooked rice contributed to the differences in stickiness. Since the stickiness measured by a texture analyser actually reflects the adhesiveness between interfaces i.e. the surface between rice kernel and TPA probe, this indicates that physical and chemical characteristics of the surface materials (the leached materials during cooking) are likely to be a major determinant of the stickiness between rice grains.

Using a set of rice cultivars differing in terms of amylose content, the objectives of this study are: 1) to identify and characterize the amounts and molecular structural features (both chain-length distribution (CLD) and molecular size, measured by size-exclusion chromatography (SEC, also termed GPC or HPLC-SEC, where the size separation parameter is the hydrodynamic radius *R*_h_) of the leached starch; and 2) to devise mechanistic reasons for any differences in terms of leaching characteristics and molecular structural features of leached starch.

## Results

### Stickiness of freshly cooked rice with and without hot-water washing

**Figure 1:**
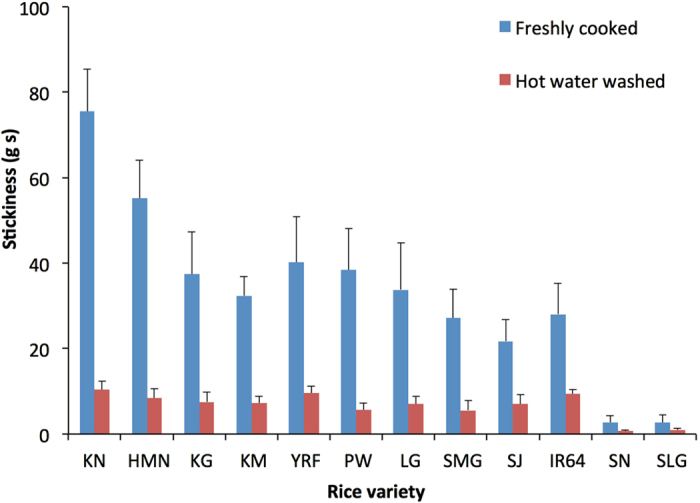
Stickiness of all rice samples measured from TPA.

The stickiness of the grains from 12 freshly cooked rice varieties displays significant differences. KN and HMN, both waxy rices, are the two stickiest, whereas SN and SLG, both high-amylose rices, show extremely low stickiness (Fig. 1). This is consistent with our previous results that stickiness is always negatively correlated with amylose content^3^,^5^. After hot-water washing, most varieties, except SN and SLG, show similar and reduced stickiness values. As displayed in Table 1, the relative stickiness loss ranges from 65 to 86%, and both the absolute and relative amounts of stickiness loss are reduced with increasing amylose content (Table 1).

### Composition of leached materials

The composition of the leached materials is presented in Table 1. The total starch content ranges from 81.2 to 92.6%, the protein content from 1.2 to 3.0%, and amylose content of the leached starch ranges from nearly 0 to 44%. Both leached starch and protein content show little significant difference while leached amylose content is significantly different. Rices with higher amylose content leach more amylose. The total solids of leached materials range from 15 to 55 mg per initial weight (g) rice kernel, making total weight of leached starch and protein significantly different between cultivars and also showing that high-amylose rices leached less material than waxy or low-amylose rices.

### The structural characterization of leached starch

**Figure 2:**
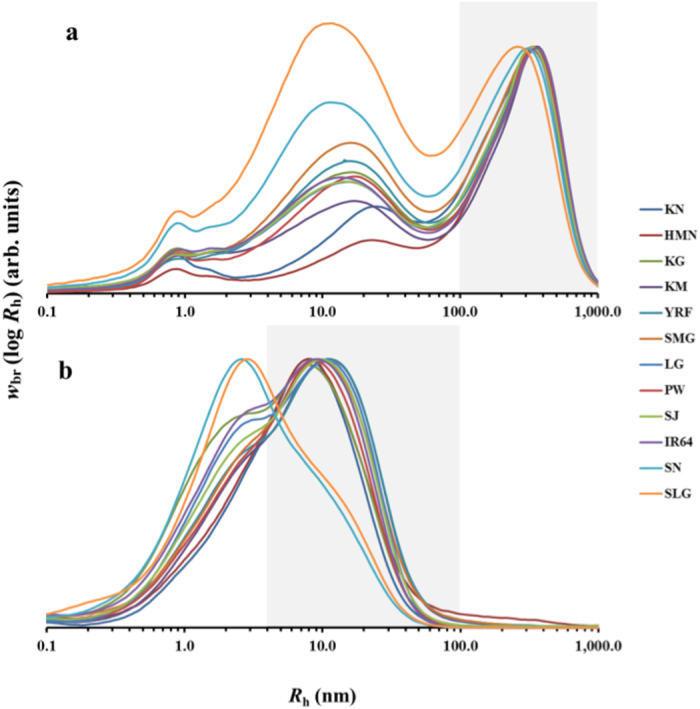
SEC weight distributions of branched starch molecules, *w*_br_(log*R*_h_), normalized to the highest peak. (**a**) Weight distributions for native grain starch. (**b**) weight distributions for leached starch. The grey area denotes the *R*_h_ range of amylopectin in native grain and leachate, respectively.

Figure 2 presents typical SEC weight distributions of branched starch molecules. As shown in Fig. 2a and described elsewhere^5^,^21^, the fully branched distributions of native grain starch show two populations of α-glucans: amylose (*R*_h_ ≤ 100 nm) and amylopectin (*R*_h_ > 100 nm). The elution pattern of the two waxy varieties indicates that there is some co-elution of small amylopectin molecules and large amylose molecules. Another small peak at *R*_h_ ~1 nm may be residual proteins, due to the incomplete hydrolysis by protease during the starch extraction procedure. For the leached starches (Fig. 2b), the molecular size distributions are over a significantly smaller range (1 ~100 nm) than those of native grain starch (1 ~1000 nm), with almost none of the very large molecules present in the leachate. There are two populations of molecules in the leached starches, at *R*_h_ ~1 nm and ~10 nm. The leached amylose and amylopectin were not clearly separated, which may either be because their ranges overlapped in size, or the limitations of SEC separation for the set-up used here. As mentioned above, waxy rice leaches mainly amylopectin, but high-amylose rice leaches significantly higher proportions of amylose. Table 2 shows the average molecular size of amylopectin and amylose, 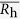, as defined elsewhere^5^. The 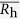
_,Ap_ of grain amylopectin is about 30 times higher than that of leached amylopectin while the 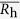
_,Am_ of grain amylose is about 5 times higher than that of leached amylose.

**Figure 3:**
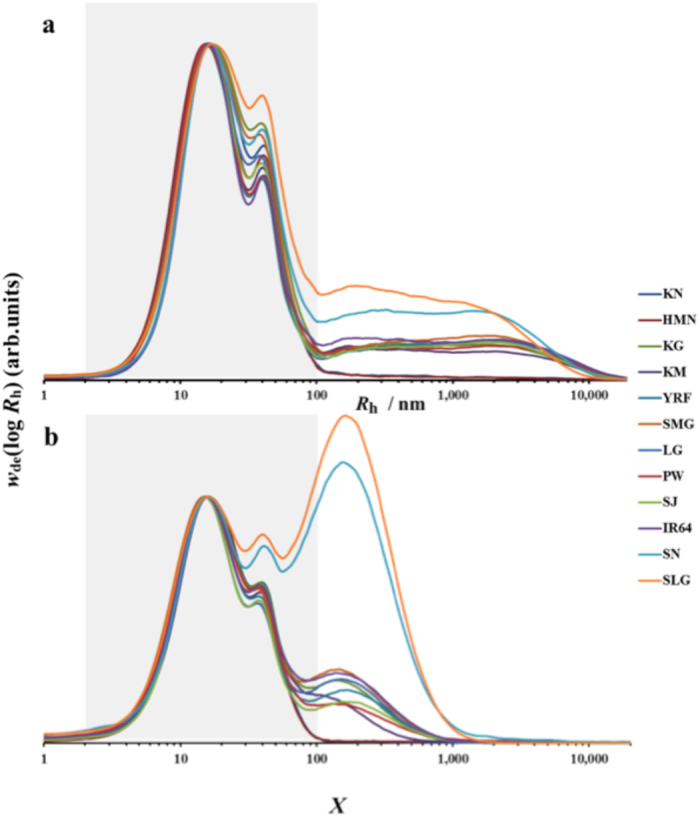
SEC weight CLDs of debranched starches. All distributions were normalized to the amylopectin peak. (**a**) Weight CLDs for native grain starch. (**b**) Weight CLDs for leached starch. The grey area denotes the *R*_h_ range of amylopectin in native grain and leachate, respectively.

Figure 3 displays typical weight chain-length distributions (CLDs) of debranched starches. The components with *X* ≤ 100 are defined as amylopectin chains, while those with *X* > 100 are defined as amylose chains^22^. For grain (Fig. 3a) and leached (Fig. 3b) starches, the weight CLDs of amylopectin show the usual features of two large Ap peaks (denoted AP1 and AP2, respectively). The waxy varieties also show the presence of some very long chains, with *X* > 100, which are absent in the CLD of the leached starch. As displayed in Table 2, for either native grain starch or leached starch, *X*_AP1_ is about DP 15–17 while *X*_AP2_ is between DP 37–40, showing little significant differences. However, as shown in Fig. 3 and Table 2, the height of the second peak (denoted *h*_AP2_) varies significantly, especially for high-amylose rices, for which *h*_AP2_ of both grain and leached starches are much higher than that of other rice samples. Table 2 also gives a subdivision method by Hanashiro *et al*.^23^ to separate the Ap region into four categories: *X* = 6–12, 13–24, 25–36, and 37–100, described as short, medium, long, and very long chains respectively. Waxy rices (KN and HMN) have more short branches and fewer very long chains, whereas high-amylose rices show an opposite distribution with less short chains but more very long chains. For the Ap CLD comparing grain and leached starch, most rice cultivars, except high-amylose rices, do not display large variations. Compared to the Ap CLD of grain starch for high-amylose rices, the leached starch of high-amylose rices contains significantly less medium and long chains but more very long chains. Even though the average DP of amylopectin 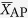 of both grain and leached starch is not significantly different (Table 2), the AM CLDs between grain and leached amylose are obviously different. The AM branches of grain starch range from DP 100 to 20,000 (Fig. 3a), whereas that of leached starch just range between DP 100 and 1000 (Fig. 3b and Table 2). Even though the amount of leached amylose varies between cultivars, the average DP (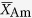) of leached amylose is not significantly different compared to that of native amylose in the grain. Interestingly, the super-long chains seen for the two waxy varieties in the CLD of the grain starch are not present in the CLD of the leached starch for those samples (Fig. 3b).

### The relation between leached materials, leached amylopectin molecules and stickiness

#### The relations between the amount and compositions of leached materials and stickiness

As shown in Table 3 and the indication from the aforementioned result, rice with a higher amount of leached materials is stickier, i.e. leached material plays a significant role in determining stickiness between grains. In Table 3, the starch and protein contents in the leached material show no significant correlations with stickiness while the total starch and protein weights in the leached material significantly correlate with stickiness. However, both the percentage and total weight of leached amylose (or amylopectin) strongly and negatively (or positively) correlate with stickiness. As displayed in Fig. 1 and Table 1, waxy rices, the stickiest rices, leach amylopectin, whereas high-amylose rices, which show extremely low stickiness, leach nearly 50% of their amylose.

#### Relations between the molecular structure of leached amylopectin and stickiness

As displayed in Table 3, both the stickiness of cooked rice with or without hot-water washing and the stickiness loss value are positively correlated with the molecular size of leached amylopectin and the proportions of amylopectin chains with DP ≤ 36, and negatively correlated with the proportion of amylopectin chains with DP > 36.

#### The effect of amylose content

As shown in Table 3, amylose content is negatively correlated with the stickiness of the freshly cooked rice, as reported elsewhere^3^,^4^,^5^. Here, it is shown for the first time that both the absolute and relative loss of stickiness by hot-water washing is negatively correlated with amylose content, meaning that rice with higher amylose content tends to reduce its stickiness to a smaller degree by hot-water washing. This can be illustrated by the negative correlation between amylose content and total amount of leached materials, i.e. that rices with higher amylose content leach less during rice cooking, thereby causing less sticky texture and less stickiness loss. Also, a significant positive correlation between amylose content and the leached amylose content is seen in Table 3, where the leached amylose content can amount to 44% of the total leached starch (Table 1). Furthermore, the amylose content correlates negatively with the molecular size and proportion of short branches of leached amylopectin (Table 3).

## Discussion

Starch is the main component of the rice grain. When rice is cooked, the main physical change is starch gelatinization. When starch granules swell as a result of the loss of the crystalline order and the absorption of water^24^, the amylose and small amylopectin molecules leach out from the granules. The leached amylose can form a three-dimensional network^25^. Initially, it was thought that amylose was the main leached component, and that it formed a three-dimensional network during cooling of the starch paste^25^. It was commonly assumed that amylose in non-waxy varieties could be separated from amylopectin by aqueous dispersion in hot water^26^,^27^,^28^. Later, SEC data showed that the water-soluble fraction of non-waxy starch generally contains both amylose and smaller amylopectin molecules^29^,^30^,^31^,^32^. In this paper we report for the first time that the molecular size of leached amylopectin is about 30 times smaller than that of grain amylopectin while the molecular size of leached amylose is about 5 times smaller than that of grain amylose, and that the CLD of leached amylopectin is similar to that of native amylopectin while that of leached amylose is over a much smaller range than that of the total amylose. Even for high-amylose rices, which leach least, the leached amylopectin content can be up to 56% of total leached starches (Table 2). In contrast to the earlier suggestion that the main leachate during gelatinization is amylose, we report that it is mainly amylopectin (at least in the varieties studied here, which cover a wide range of amylose content).

It is reported that the CLDs of amylopectin are independent of molecular size^33^,^34^. Here we also find the leached amylopectin with much smaller molecular size has a similar CLD to the native amylopectin in the region between DP 6 to 100. This study further proves that amylopectin molecules have a wide size distribution. The varieties shown in this study range in amylose content, and the region between *R*_h_ 10–100 nm is where amylose molecules elute (Fig. 2a). However inspection of the elution profile of the two waxy varieties shows very clearly that there are small amylopectin molecules that co-elute over the whole range of the amylose molecules, indicating that the peak spanning *R*_h_ ~3–100 nm consists of both amylose and small amylopectin molecules in the non-waxy varieties, and small amylopectin molecules in the waxy varieties. Comparing the elution profiles in Fig. 2, it is clear that the amylopectin component of the leachate consists of the small amylopectin molecules. Furthermore, the leached amylopectin molecules have a smaller average chain length than the amylopectin molecules from total starch, and they have fewer chains with *X* > 36 (Table 3), which span and carry multi-clusters^35^. Together those data suggest that the smaller amylopectin molecules have fewer clusters, and fewer chains that span multiple clusters.

Another noteworthy point, as shown in Fig 3a, is that native waxy starch always has a small amount of very long chains that elute in the region where amylose is usually found (up to about DP 3000), but the longest chains in the leachate of the waxy rice is about at DP 100, consistent with a previous study. It has been found that different fractions of size-separated amylopectin have similar CLDs of all but the longest chains^35^, as also seen here, but there is a distinct difference in the CLD of the very long chains. Together with the fact that the average molecular size of the native amylopectin is about 30 times that of the leached one, we can infer that these very long amylopectin chains are the C chains which carry other short chains i.e. A- and B-chains, and span multi-clusters (more than 4), thereby contributing a significantly high molecular size, or as chains that surround and define structures such as blocklets, which are seen as the very large molecules in Fig. 2a. Even when starch is gelatinized in ordinary cooking methods (as done here), there are still water-insoluble large molecules and often some residual crystallinity. In the present work, smaller amylopectin molecules are seen to be soluble, and we speculate that these small amylopectin molecules are not linked to the main blocklet structure and are thus free to leach upon gelatinization. Consistent with this, a previous study^36^ showed that the amount of leachate from waxy rices increased with heating across the gelatinisation endotherm, and reached a plateau at higher temperature and long heating. Thus, we can infer that, in the native starch granules, the small amylopectin molecules may entangle with large amylopectin molecules by non-covalent bonding or co-crystallize with other large amylopectin molecules, and these small ones in the leachate may be located at the edges of blocklets, and are free to leach once the crystalline structure is destroyed by heating. Therefore, the data presented here provides a lens into the structural organisation of starch that enables a molecular explanation for the observation of small amylopectin molecules causing stickiness.

It is shown here and elsewhere^20^ that stickiness increases with the total amount of leached materials and the content of leached amylopectin. Branched polymers typically exhibit shear and extensional viscosities that are unobtainable with linear polymers^37^. At low shear rates, a branched polymer can exhibit a viscosity two orders of magnitude greater than that of linear polymers of the same molecular weight^38^. This is why a starch paste with higher amylopectin content always displays more viscous and less elastic rheological properties, while amylose molecules act as a diluent in terms of viscous properties^39^. It has been shown, e.g. in the 2-dimensional data of Vilaplana *et al*.^40^, that starches with higher amylose content have significant amounts of material intermediate between amylopectin and amylose in structural characteristics; these could be a component of the leached material. However, the amount of leached amylopectin is not the only reason contributing to the stickiness between cooked rice grains. It is seen here for the first time that the stickiness between cooked rice kernels is also governed by molecular size and chain length of the leached amylopectin, i.e. the more short chains, the bigger molecular size of leached amylopectin, and the greater the stickiness between cooked rice grains. For synthetic polymers, the solution rheology is strongly influenced by molecular size and branching structure. For branched polymers, chain crowding and interpenetration also constrain chain motion, thereby causing a higher viscous resistance than that of linear polymers^38^. Thus a higher degree of branching (as seen here in the ratio of short AP (*X* ≤ 36) to long AP (*X* > 37) chains), together with larger molecular size of leached amylopectin, produces a higher viscous resistance, i.e. a higher stickiness.

In this study, the way that TPA measures the property termed “stickiness” (which follows the same principle as that of measuring tack in adhesion) is as follows. A single layer of cooked rice grains is placed on a baseplate. A two-cycle force/distance compression test is conducted with a probe which descends slowly (step 1: bond formation) and then is moved back (step 2: bond separation)^6^. Both of these clearly relate to how the human mouth would perceive the stickiness of a material to tongue and teeth during the first chew, which explains the TPA/panel data correlations^3^. The quantity defined as TPA stickiness depends on a number of fundamental properties, including the bulk viscosity^41^.

**Figure 4:**
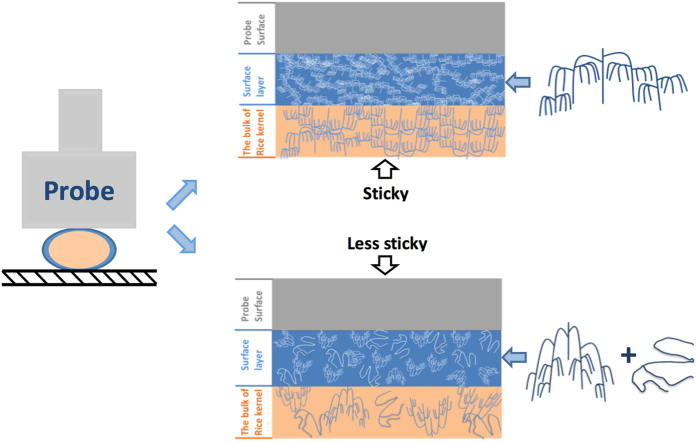
The postulated molecular mechanism for stickiness between cooked rice grains and the TPA probe. The surface layer of the sticky one has more amylopectin molecules with higher proportion of short chains with DP ≤ 36 and bigger molecular size, while the surface layer of the less sticky one has less amylopectin molecules (diluted by amylose molecules), fewer short chains with DP ≤ 36, and smaller molecular size.

Since TPA stickiness is the resistance offered by the cooked rice grains to detachment from the probe, the higher the stickiness value, the more force is needed to make the grains and probe come apart. As presented in section 3.4, in the leachate, the stickiness increases with increasing total amount of amylopectin, the proportion of short amylopectin chains with DP ≤ 36, and amylopectin molecular size. Figure 4 shows the postulated mechanism for stickiness between cooked rice grains and the probe. There is an interface of leachate connecting the grains and the probe. Larger amylopectin molecules with higher proportions of short branches (DP ≤ 36) in the leachate can adhere to more area on the probe surface, and thus provide better bonding. On the other hand, these larger amylopectin molecules also interact with other amylopectin molecules in the leachate and in the bulk of rice kernel by H bonding, which creates viscous resistance to the detachment from the probe. The overall molecular mechanism involves H bonding between the leached small amylopectin molecule and probe, between amylopectin molecules in the leachate, and between the leached amylopectin and the bulk of the grain. An increase of the amount of amylopectin, the proportion of short amylopectin chains, and amylopectin molecular size all create a greater opportunity for bonding and molecular interaction, i.e. a higher stickiness value.

A significant effect of amylose content (arising from cultivar differences) on leaching characteristics is also seen, which could be an underlying cause of the stickiness difference between rice varieties. As shown in Table 3, the leached amylopectin content, the total amount of leached materials and the molecular size of leached amylopectin both decrease with increased amylose content. This is probably because amylose molecules are more likely to span multiple crystalline-amorphous lamellae in the grain, and to participate in the crystallization of amylopectin branches, which would restrict the starch swelling and leaching during heating.

Previous studies showed that the amount of leached amylose depends on the total amylose content^2^,^29^, and that amylose content positively correlates with hardness and negatively correlates with stickiness^2^,^3^,^5^. As shown in Table 2 and Fig. 4, not only the stickiness, the leached amylose content and the total amount of leached materials, but also the molecular structural features of leached starch are also associated with amylose content. The limited swelling causes a reduction in the amount of leached materials (mainly amylopectin) and ultimately gives rise to a harder rice texture after cooking; the smaller amount of leached amylopectin, and the smaller molecular size and proportion of short branches of leached amylopectin in these cases, also contribute to a less sticky texture.

## Conclusion

This study reveals that stickiness between cooked rice grains is determined by the total amount, molecular size and chain structure (CLD) of leached amylopectin. We present the first unified molecular-based mechanistic description of the causes of these important sensory properties, using the results in this study and previous findings by ourselves^3^,^5^ and others^2^,^4^,^29^. Starches with certain structural features can leach from rice kernels during cooking and attach on the surface of the cooked rice grains. The molecular size of leached amylopectin is about 30 times smaller than that of native amylopectin, while that of leached amylose is about 5 times smaller than that of grain amylose. Leached amylopectin has a similar CLD to that in the grain, while the leached amylose branches have smaller chain lengths, mainly between DP 100–1000. The postulated mechanism for stickiness between cooked rice grains and the probe is that an increase of the amount of amylopectin, the proportion of short amylopectin chains, and amylopectin molecular size in the leachate all create a greater opportunity for bonding and molecular interaction, causing more force to be needed to make the grains and probe come apart, i.e. a higher stickiness value.

An underlying origin of the stickiness differences between rice cultivars is the amylose content in the whole grain starch. With increasing amylose content, the total amount of leached materials, the amylopectin content in the leachate, and the molecular size and the proportion of short branches of leached amylopectin, all decrease, leading to a lower stickiness. However, amylose content is not the sole determinant. In some cases, amylose content is similar but the hardness^5^ and/or stickiness^20^ still vary significantly. This is because of the effects of other structural features. One such is amylose chain-length distributions. Our previous finding points out that high-amylose rice tends to have higher proportions of short amylose chains^5^. Whether this is a characteristic of all high-amylose rices could provide insight into their functional differences. Another determining structural feature is the interaction between amylose and amylopectin molecules (the location of amylose) in native starch granules. The location of amylose in native starch granules is not completely understood, but it is often thought that amylose molecules are present in an amorphous conformation^42^,^43^; further, there are suggestions that amylose is spread among amylopectin crystallites^5^,^44^, and may co-crystallize with amylopectin chains.

By quantifying the components and the molecular structure of leached starch, rice breeders could choose lines which optimize the texture of cultivars. For example, a cultivar which leaches more amylopectin with more short amylopectin chains and bigger molecular size would be sticky after cooking, which could be desirable for sushi. On the other hand, a cultivar which leaches more amylose should be less sticky but have a harder texture. This molecular structural mechanism provides a new tool for rice breeders to select cultivars with desirable palatability.

## Materials and Methods

### Materials

Twelve varieties of rice were selected with a wide range with known phenotypes and genotypes for quality traits (Table 1). After harvesting, all rice samples were dehulled in a dehusker (Otake, Aichi, Japan), polished to yield rice with the same whiteness value in a commercial mill (FASCO, VIC, Australia), and then stored in self-sealing plastic bags in a refrigerator prior to analyses.

Protease from *Streptomyces griseus* (type XIV), and LiBr (ReagentPlus) were purchased from Sigma-Aldrich Pty. Ltd. (Castle Hill, Australia). Isoamylase (from *Pseudomonas sp.*) and a D-glucose (glucose oxidase/peroxidase; GODOP) assay kit were purchased from Megazyme International, Ltd. (Wicklow, Ireland). A series of pullulan standards with peak molecular weights ranging from 342 to 2.35 × 10^6^ were from Polymer Standards Service (PSS) GmbH (Mainz, Germany). Dimethyl sulfoxide (DMSO, GR grade for analysis) was from Merck Co. Inc. (Kilsyth, Australia). All other chemicals were reagent-grade and used as received.

### Rice cooking

Before cooking, residual bran and other adhering powders were removed from white rice kernels with an aspirating device. A 10-g sample of white rice was placed in a 100 mL beaker, and distilled water was added to the rice to give a rice-to-water weight ratio of 1:1.6. Thereafter, the beaker was sealed with aluminium foil, placed on a steaming tray, and cooked in a household rice cooker (Kambrook Rice Express, VIC, Australia) for 30 min.

### Texture profile analysis (TPA)

After cooking and cooling to room temperature, a 1-g subsample of cooked rice grains was weighed and placed as a single layer of grains on the base plate. A two-cycle, force-versus-distance compression program was used for measurements with a TA.XT-Plus Texture analyser with a 35 mm cylindrical probe attachment (Stable Micro Systems Ltd., Surrey, UK). The probe descended at a speed of 1 mm/s, returned, and then the compression cycle was repeated. Compression was set to 40% strain to avoid destroying the rice grain. For each of 3 cooking replicates, texture measurements were conducted 6 times on the 1-g subsample of cooked rice grains. Stickiness between grains was recorded as the area of the negative force curve.

### Extraction of leached materials

A sample of white rice (10 g) was cooked as described above. The leached materials on the surface of the cooked rice were extracted by rinsing with 100 mL of hot deionized water (~95 °C) with very gentle stirring using a glass rod for 5–10 s before filtering through a 250 *μ*m sieve. The rinsing procedure was repeated again with 50 mL of hot deionized water. Both the washed kernels and the rinsing water were retained. The rinsed rice kernels were cooled and used to measure stickiness again by TPA. The water was frozen immediately using liquid nitrogen, and then freeze-dried for storage and further analysis. The total weight of the leached materials was recorded after freeze-drying.

### Composition analysis of leached materials

Total starch content of leached materials was measured using a Megazyme total starch (AA/AMG) assay kit following a method described elsewhere^45^. The protein content of leached materials was determined using a BCA Protein Assay Kit (Pierce).

### Molecular size distributions of both whole-grain starch and leached starch molecules

The structure of extracted whole starch and leached starch molecules was characterized by SEC using an Agilent 1100 Series SEC system (Agilent Technologies, Waldbronn, Germany) equipped with GRAM 30 and 3000 analytical columns (PSS) and a refractive index (RI) detector (RID-10A, Shimadzu Corp., Kyoto, Japan), following a method described elsewhere^46^,^47^. The molecular size distribution of branched starch was plotted as the SEC weight distribution, *w*_br_(log*R*_h_). For branched starch molecules, as for any branched polymer, there is no unique relation between size and the molecular weight. For the debranched samples, which are linear, the relation between *R*_h_ and molecular weight *M* was obtained as follows. The assumption of universal calibration for SEC is that the elution time of the analyte depends only on its *R*_h_ and not on its structure, whence one has for two linear polymers, a sample and a standard, the relation:


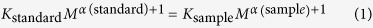


where *K* and α are the Mark-Houwink parameters for the polymer, solvent and temperature being used. Pullulan standards with known peak molecular weights were used for calibration to obtain a relationship between SEC elution volume and *R*_h_ of starch molecules following the Mark-Houwink equation:


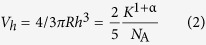


Here *N*_A_ is Avogadro’s constant. The Mark-Houwink parameters *K* and *α* of pullulan in DMSO/LiBr solution at 80 °C are 2.424 × 10^−4^ dL g^−1^ and 0.68, respectively^46^.

### Starch debranching and measurement of CLD of debranched starch by SEC

The extracted starch (~4 mg) was dissolved in 0.9 mL of deionized water and then mixed with 2.5 *μ*L isoamylase (1000 U mL^−1^), 0.1 mL acetate buffer solution (0.1 M, pH 3.5), and 5 *μ*L sodium azide solution (0.04 g mL^−1^). The mixture was incubated at 37 °C for 3 h. The debranched starch suspension was then heated in a water bath at 80 °C for 2 h after being neutralized with 0.1 M NaOH solution, and then freeze-dried overnight. The dried debranched starch was dissolved in DMSO/LiBr (0.5%) solution for SEC analysis.

To obtain SEC distributions of debranched starch, GRAM 100 and GRAM 1000 columns (PSS) were used, with the same pullulan standards and procedure as that used to calibrate the SEC for whole branched molecules. The SEC weight distribution, *w*(log*X*), obtained from the DRI signal was plotted against *X* (degree of polymerization DP), with *X* being determined using the Mark-Houwink relationship (see Equation 1) and with *M* = 162.2(*X*–1) + 18.0 (162.2 is the molecular weight of the anhydroglucose monomeric unit and 18.0 is that of the additional water in the end groups); *K* and *α* for linear starch chains in the eluent of DMSO/LiBr at 80 °C are 1.5 × 10^–4^ dL g^–1^ and 0.743, respectively^5^. For a linear polymer (such as debranched starch), the number distribution (obtained by debranching), *N*_de_(*X*), is related to the corresponding weight distribution by^48^:


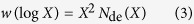


The amylose content of all rices was determined from the SEC weight distributions of debranched starch following the procedure described by Syahariza *et al*.^21^. This method has been shown to be more accurate than the iodine colorimetric method^22^.

### Statistical analysis

For each structural measurement, duplicate analyses were performed for each sample. All data were reported as mean ± standard deviation (SD) using analysis of variance (ANOVA) with Tukey’s pairwise comparisons. Significant differences of the mean values were determined at *p* < 0.05. The textural measurements were analyzed in duplicate for each sample. One-way analysis of variance (ANOVA) and both Pearson and Spearman rank correlation methods were carried out using SPSS V. 16.0 software (SPSS Inc., Chicago, IL). The means of duplicated measurements were used for the correlation analysis.

## Additional Information

**How to cite this article**: Li, H. *et al*. The molecular structural features controlling stickiness in cooked rice, a major palatability determinant. *Sci. Rep.* **7**, 43713; doi: 10.1038/srep43713 (2017).

**Publisher's note:** Springer Nature remains neutral with regard to jurisdictional claims in published maps and institutional affiliations.

## Figures and Tables

**Table 1:** Parameters of the stickiness and the leaching characteristics of all rice varieties.

Rice variety	Abbreviation code	Country of origin	Amylose content	Stickiness (g∙s)		Stickiness Loss	
				Freshly cooked	Hot-water washed	Absolute (g∙s)	Relative
Khao Niao	KN	Thailand	3.3 ± 0.1% a	75.4 ± 22.3 e	10.3 ± 2.1 d	65	86%
Hom mali Niaw	HMN	Australia	2.6 ± 0.3% a	55.2 ± 8.9 d	8.4 ± 2.1 b–d	47	85%
Kangaroo	KG	Australia	19.6 ± 0.9% b	37.4 ± 9.8 b,c	7.5 ± 2.2 b–d	30	80%
Kyeema	KM	Australia	19.5 ± 0.5% b	32.4 ± 4.6 b,c	7.3 ± 1.5 a,b	25	78%
YRF209	YRF	Australia	22.3 ± 0.2% b,c	40.1 ± 10.7 c,d	9.6 ± 1.6 c,d	31	76%
Pandan Wangi	PW	Australia	20.4 ± 1.9% b	38.4 ± 9.7 c	5.6 ± 1.6 b	33	85%
Langi	LG	Australia	21.7 ± 0.1% b,c	33.8 ± 11.0 b,c	6.9 ± 1.9 a,b	27	79%
Sunrice Medium Grain	SMG	Australia	21.8 ± 0.9% b,c	27.2 ± 6.7 b,c	5.6 ± 2.3 b	22	80%
Sunrice Jasmine	SJ	Australia	21.2 ± 1.4% b	21.7 ± 5.0 b	7.0 ± 2.2 a,b	15	68%
IR64	IR64	Australia	24.9 ± 0.5% c	28.1 ± 7.3 b,c	9.4 ± 0.9 c,d	19	66%
Swarna	SN	India	31.2 ± 0.1% d	2.7 ± 1.5 a	0.6 ± 0.2 a	2	76%
Sunrice Long Grain	SLG	Thailand	32.0 ± 0.2% d	2.7 ± 1.8 a	0.9 ± 0.4 a	2	65%
	**Components content (%) in the leachate**		**Amylose content of the leached starch**	**Total solids of the leachate (mg/g rice kernel)**	**Components weight in the leachate (mg/g rice kernel)**		
	**Starch**	**Protein**			**Total starch**	**Total amylose**	**Total protein**
KN	89.64 ± 0.00 a,b	2.96 ± 0.00 b	0.72 ± 0.00 a	43.7 ± 0.00 g	33.7 ± 0.00 f	0.2 ± 0.00 a	1.1 ± 0.00 d
HMN	89.67 ± 0.64 a,b	2.01 ± 0.06 a,b	1.73 ± 0.17 a	55.1 ± 2.79 h	42.5 ± 2.45 g	0.7 ± 0.12 a,b	1.0 ± 0.08 c,d
KG	91.23 ± 3.36 a,b	2.58 ± 1.17 a,b	15.52 ± 0.34 b,c	23.0 ± 0.00 b,c	18.1 ± 0.67 b,c	2.8 ± 0.16 c,d	0.5 ± 0.23 b,c
KM	91.30 ± 5.72 a,b	1.91 ± 0.04 a,b	10.26 ± 2.21 b	25.2 ± 1.19 c,d	19.8 ± 2.17 b–d	2.1 ± 0.66 b,c	0.4 ± 0.01 b,c
YRF	92.39 ± 1.26 b	1.55 ± 0.27 a,b	14.66 ± 0.69 b,c	27.7 ± 1.19 d,e	22.0 ± 1.24 c–e	3.2 ± 0.33 c,d	0.4 ± 0.05 a,b
PW	92.65 ± 4.36 b	2.24 ± 0.07 a,b	11.63 ± 1.74 b,c	33.2 ± 0.35 f	26.5 ± 0.97 e	3.1 ± 0.35 c,d	0.6 ± 0.01 b,c
LG	91.76 ± 0.63 a,b	2.13 ± 0.59 a,b	17.07 ± 0.44 b,c	30.3 ± 0.08 e,f	23.9 ± 0.10 d,e	4.1 ± 0.12 d,e	0.6 ± 0.16 b,c
SMG	90.08 ± 0.30 a,b	2.57 ± 0.32 a,b	17.93 ± 4.89 c	23.8 ± 0.85 b–d	18.4 ± 0.60 b,c	3.3 ± 0.79 c,d	0.5 ± 0.08 b,c
SJ	81.18 ± 2.83 a	2.67 ± 0.16 a,b	13.04 ± 0.57 b,c	22.7 ± 0.45 b,c	15.9 ± 0.87 a,b	2.1 ± 0.02 b,c	0.5 ± 0.04 b,c
IR64	90.97 ± 2.57 a,b	1.16 ± 0.11 a	18.17 ± 1.81 c	26.0 ± 0.59 c–e	20.3 ± 0.11 b–d	3.7 ± 0.35 c,d	0.3 ± 0.02 a
SN	86.40 ± 0.54 a,b	2.02 ± 0.27 a,b	42.61 ± 0.00 d	15.8 ± 1.46 a	11.7 ± 1.16 a	5.34 ± 0.00 e	0.3 ± 0.06 a
SLG	90.93 ± 2.43 a,b	1.96 ± 0.03 a,b	44.05 ± 2.36 d	20.3 ± 0.58 b	15.9 ± 0.88 a,b	7.0 ± 0.76 f	0.3 ± 0.00 a,b

Absolute value of stickiness is calculated by the stickiness of freshly cooked rice minus that of hot-water washed rice; The absolute value of stickiness loss relative to the stickiness of the freshly cooked rice.

**Table 2:** Starch molecular parameters extracted from SEC for all native grain starches and leached starches.

		Amylopectin								
Rice variety & treatment		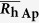	*X*_Ap1_	*X*_Ap2_	*h*_Ap2_	6 < *X* ≤ 12	12 < *X* ≤ 24	24 < *X* ≤ 36	36 < *X* ≤ 100	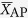
Grain starch	KN	223.5 ± 4.4^b,c^	16.1 ± 0.0^a–c^	40.9 ± 0.0^e^	0.70 ± 0.00^c–f^	24.3 ± 0.0%^a–e^	37.4 ± 0.0%^c–f^	16.9 ± 0.0%^d–h^	21.4 ± 0.0%^a–e^	14.6 ± 0.0^a^
	HMN	242.0 ± 0.9^c^	15.2 ± 0.8^a,b^	40.4 ± 1.7^d,e^	0.67 ± 0.01^b–f^	26.8 ± 3.1%^c–e^	37.1 ± 1.2%^c–f^	15.6 ± 0.6%^a–f^	20.5 ± 1.2%^a–e^	13.6 ± 1.6^a^
	KG	241.8 ± 10.7^c^	17.2 ± 1.5^b,c^	39.0 ± 0.7^a–e^	0.76 ± 0.08 ^f–h^	22.2 ± 2.5%^a–c^	36.2 ± 1.0%^b–e^	17.9 ± 1.3%^h^	23.7 ± 2.2%^e,f^	14.0 ± 2.2^a^
	KM	248.5 ± 3.4^c^	16.3 ± 0.3^a–c^	40.0 ± 0.7^b–e^	0.63 ± 0.00^a–d^	22.6 ± 1.0%^a–d^	39.7 ± 0.4%^c–f^	16.0 ± 0.2%^b–f^	21.8 ± 0.4%^a–e^	13.9 ± 0.4^a^
	YRF	235.4 ± 3.8^c^	15.4 ± 0.3^a,b^	40.0 ± 0.2^b–e^	0.60 ± 0.01^a,b^	26.9 ± 1.4%^c–e^	38.6 ± 0.6%^c–f^	15.1 ± 0.3%^a–c^	19.3 ± 0.5%^a–c^	13.8 ± 1.6^a^
	PW	224.9 ± 13.5^b,c^	15.4 ± 0.3^a,b^	40.2 ± 0.5^c–e^	0.61 ± 0.00^a–c^	26.1 ± 1.1%^c–e^	38.3 ± 0.2%^c–f^	15.2 ± 0.1%^a–c^	20.4 ± 0.8%^a–e^	14.5 ± 0.2^a^
	LG	232.5 ± 17.0^c^	16.3 ± 0.0^a–c^	38.3 ± 1.2^a–e^	0.67 ± 0.06^b–f^	22.5 ± 0.5%^a–d^	39.1 ± 1.4%^d–f^	17.2 ± 0.8%^e–h^	21.2 ± 1.2%^a–e^	14.6 ± 0.6^a^
	SMG	241.2 ± 11.9^c^	16.5 ± 0.0^a–c^	38.0 ± 0.2^a–e^	0.73 ± 0.01^d–g^	23.8 ± 0.2%^a–e^	36.8 ± 0.8%^b–f^	17.7 ± 0.2%^g,h^	21.8 ± 0.8%^a–e^	13.2 ± 2.6^a^
	SJ	233.4 ± 10.8^c^	16.1 ± 0.0^a–c^	39.2 ± 0.5^a–e^	0.65 ± 0.01^a–d^	22.9 ± 0.2%^a–d^	39.4 ± 0.1%^e,f^	16.6 ± 0.2%^c–h^	21.2 ± 0.3%^a–e^	14.2 ± 0.5^a^
	IR64	244.6 ± 14.4^c^	16.0 ± 0.1^a–c^	40.9 ± 0.5^e^	0.61 ± 0.01^a–c^	23.6 ± 0.2%^a–e^	39.6 ± 0.0%^f^	15.0 ± 0.1%^a–c^	21.8 ± 0.1%^a–e^	14.7 ± 0.2^a^
	SN	210.0 ± 11.9^b,c^	16.8 ± 0.4^a–c^	39.8 ± 1.0^b–e^	0.75 ± 0.02^e–g^	20.5 ± 0.9%^a,b^	36.3 ± 0.1%^b–f^	17.3 ± 0.8%^f–h^	25.9 ± 0.2%^f,g^	14.6 ± 1.3^a^
	SLG	190.4 ± 2.4^b^	17.5 ± 1.1^c^	39.8 ± 0.0^b–e^	0.85 ± 0.03^h^	20.5 ± 0.6%^a,b^	33.5 ± 0.4%^b^	17.7 ± 0.4%^h^	28.3 ± 0.6%^g^	15.1 ± 0.7^a^
Leachedstarch	KN	8.7 ± 0.1^a^	16.0 ± 0.1^a–c^	39.5 ± 0.0^a–e^	0.65 ± 0.00^a–e^	26.4 ± 0.0%^c–e^	37.9 ± 0.2%^c–f^	16.7 ± 0.1%^c–h^	19.1 ± 0.3%^a,b^	11.0 ± 1.2^a^
	HMN	9.1 ± 0.2^a^	15.7 ± 0.1^a–c^	39.0 ± 0.2^a–e^	0.63 ± 0.01^a–d^	26.9 ± 0.1%^c–e^	38.2 ± 0.2%^c–f^	16.5 ± 0.0%^c–h^	18.5 ± 0.1%^a^	13.2 ± 0.1^a^
	KG	8.6 ± 0.1^a^	15.9 ± 0.0^a–c^	38.7 ± 0.2^a–e^	0.65 ± 0.01^a–e^	24.7 ± 0.0%^b–e^	36.4 ± 0.1%^b–f^	16.0 ± 0.1%^b–g^	22.8 ± 0.0%^c–f^	13.1 ± 0.8^a^
	KM	8.9 ± 0.1^a^	16.0 ± 0.1^a–c^	38.5 ± 0.0^a–e^	0.61 ± 0.02^a–c^	24.7 ± 0.3%^b–e^	37.5 ± 1.1%^c–f^	15.6 ± 0.0%^a–e^	22.1 ± 0.8%^b–e^	11.2 ± 2.6^a^
	YRF	9.0 ± 0.1^a^	15.4 ± 0.0^a,b^	37.0 ± 0.2^a–c^	0.60 ± 0.02^a,b^	27.3 ± 0.9%^d,e^	37.4 ± 0.9%^c–f^	15.4 ± 0.0%^a–d^	19.9 ± 1.7%^a–d^	11.7 ± 0.4^a^
	PW	9.1 ± 0.0^a^	15.5 ± 0.4^a–c^	38.5 ± 0.0^a–e^	0.63 ± 0.01^a–c^	27.1 ± 1.0%^d,e^	36.8 ± 1.8%^b–f^	15.9 ± 0.2%^b–f^	20.2 ± 1.1%^a–e^	10.7 ± 3.0^a^
	LG	8.5 ± 0.0^a^	15.1 ± 0.4^a^	36.7 ± 1.6^a,b^	0.57 ± 0.00^a^	27.2 ± 1.7%^d,e^	37.5 ± 0.9%^c–f^	14.8 ± 0.3%^a,b^	20.5 ± 0.5%^a–e^	11.0 ± 1.4^a^
	SMG	8.9 ± 0.2^a^	14.9 ± 0.4^a^	36.4 ± 2.0^a^	0.63 ± 0.01^a–c^	27.9 ± 1.3%^d,e^	35.3 ± 1.1%^b,c^	15.1 ± 0.1%^a–c^	21.7 ± 0.1%^a–e^	11.8 ± 0.4^a^
	SJ	8.7 ± 0.0^a^	15.2 ± 0.5^a,b^	37.5 ± 0.9^a–d^	0.58 ± 0.00^a,b^	27.1 ± 1.9%^d,e^	38.5 ± 1.1%^c–f^	15.2 ± 0.3%^a–c^	19.2 ± 0.5%^a,b^	11.9 ± 1.3^a^
	IR64	8.6 ± 0.3^a^	15.6 ± 0.5^a–c^	38.0 ± 1.2^a–e^	0.64 ± 0.01^a–d^	25.5 ± 1.2%^c–e^	35.9 ± 1.4%^b–d^	15.3 ± 0.2%^a–c^	23.4 ± 0.4%^d–f^	11.3 ± 1.4^a^
	SN	7.5 ± 0.1^a^	15.8 ± 0.0^a–c^	40.9 ± 0.0^e^	0.80 ± 0.00 ^g,h^	19.6 ± 0.0%^a^	29.2 ± 0.0%^a^	14.0 ± 0.0%^a^	37.3 ± 0.0%^h^	11.5 ± 0.0^a^
	SLG	7.6 ± 0.0^a^	15.9 ± 0.8^a–c^	40.3 ± 0.2^c–e^	0.85 ± 0.04 ^h^	20.5 ± 1.6%^a,b^	27.7 ± 0.5%^a^	14.2 ± 0.2%^a^	37.6 ± 1.9%^h^	11.5 ± 0.7^a^
**Rice variety & treatment**		**Amylose**								
		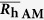	**AM content**	**100 < *****X***** ≤ 1000**	**1000 < *****X***** ≤ 20000**	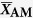				
Grain starch	KN	—	—	—	—	—				
	HMN	—	—	—	—	—				
	KG	10.6 ± 0.1^b–e^	19.6 ± 0.9%^e–g^	13.8 ± 0.5%^a–d^	5.8 ± 0.4%^b^	701.5 ± 23.9^c–e^				
	KM	10.7 ± 0.1^c–e^	19.5 ± 0.5%^e–g^	13.8 ± 0.4%^a–d^	5.7 ± 0.1%^b^	651.9 ± 2.4^c,d^				
	YRF	10.8 ± 0.1^d–e^	22.3 ± 0.2%^g,h^	14.7 ± 0.1%^b–d^	7.6 ± 0.2%^c^	805.4 ± 11.3^e,f^				
	PW	10.9 ± 0.0^d–e^	20.4 ± 1.9%^f–h^	13.9 ± 1.2%^a–d^	6.5 ± 0.7%^b,c^	727.3 ± 13.4^d–f^				
	LG	10.5 ± 0.1^b–e^	21.7 ± 0.0%^g,h^	14.3 ± 0.3%^b–d^	7.4 ± 0.4%^c^	821.3 ± 31.3 ^f^				
	SMG	11.2 ± 0.1^e^	21.8 ± 1.0%^g,h^	14.9 ± 0.6%^b–d^	6.9 ± 0.4%^b,c^	777.4 ± 32.9^e,f^				
	SJ	10.5 ± 0.2^b–e^	21.2 ± 1.4%^f–h^	14.5 ± 1.6%^b–d^	6.7 ± 0.2%^b,c^	768.6 ± 96.0^e,f^				
	IR64	10.3 ± 0.4^b–d^	24.9 ± 0.5%^h^	17.1 ± 0.2%^c,d^	7.8 ± 0.3%^c^	699.6 ± 9.0^c–e^				
	SN	10.1 ± 0.1^b,c^	31.2 ± 0.1%^i^	23.7 ± 0.6%^e^	7.5 ± 0.5%^c^	609.0 ± 37.6^b,c^				
	SLG	10.0 ± 0.1^b^	32.0 ± 0.2%^i^	26.4 ± 0.5%^e^	5.6 ± 0.6%^b^	498.2 ± 25.6^b^				
Leachedstarch	KN	—	—	—	—	—				
	HMN	—	—	—	—	—				
	KG	2.9 ± 0.5^a^	15.5 ± 0.3%^b–e^	15.3 ± 0.3%^b–d^	0.3 ± 0.1%^a^	205.6 ± 8.6^a^				
	KM	2.6 ± 0.1^a^	10.3 ± 2.2%^a^	9.9 ± 2.3%^a^	0.4 ± 0.1%^a^	192.2 ± 9.6^a^				
	YRF	2.8 ± 0.1^a^	14.7 ± 0.7%^a–d^	14.6 ± 0.7%^b–d^	0.0 ± 0.0%^a^	209.9 ± 9.5^a^				
	PW	2.9 ± 0.0^a^	11.6 ± 1.7%^a,b^	11.2 ± 1.4%^a,b^	0.5 ± 0.4%^a^	238.0 ± 15.7^a^				
	LG	2.6 ± 0.1^a^	17.1 ± 0.4%^c–f^	17.0 ± 0.5%^c,d^	0.1 ± 0.1%^a^	204.2 ± 1.9^a^				
	SMG	2.8 ± 0.2^a^	14.5 ± 0.0%^a–d^	14.1 ± 0.0%^a–d^	0.4 ± 0.0%^a^	209.5 ± 23.5^a^				
	SJ	2.8 ± 0.0^a^	13.0 ± 0.6%^a–c^	12.9 ± 0.7%^a–c^	0.1 ± 0.1%^a^	218.8 ± 1.4^a^				
	IR64	2.9 ± 0.2^a^	18.2 ± 1.8%^d–g^	17.9 ± 1.9%^d^	0.3 ± 0.1%^a^	213.1 ± 17.7^a^				
	SN	2.8 ± 0.2^a^	42.6 ± 0.0%^j^	42.0 ± 0.0%^f^	0.6 ± 0.0%^a^	220.3 ± 0.0^a^				
	SLG	2.7 ± 0.0^a^	44.1 ± 2.4%^j^	44.0 ± 2.3%^f^	0.0 ± 0.0%^a^	211.5 ± 16.4^a^				

Mean ± SD is calculated from duplicate measurements. Values with different letters in the same column are significantly different with *p* < 0.05.

**Table 3:** Correlation analysis between stickiness and leaching parameters.

Pearson Correlations																					
	Stickiness (Freshly cooked)	Stickiness (Hot-water washed)	Stickiness Loss Value	Stickiness Loss Rate	Am content	Leached materials						Molecular parameters of Leached Ap									
						Starch Cont	Am Cont	Protein Cont	Total solids	Starch weight	Am weight	Protein weight	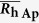	*X*_Ap1_	*X*_Ap2_	*h*_Ap2_	6 < X ≤ 12	12 < X ≤ 24	24 < X ≤ 36	36 < X ≤ 100	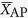
Stickiness (Freshly cooked)																					
Stickiness (Hot-water washed)	0.83**																				
Stickiness Loss Value	0.99**	0.76**																			
Stickiness Loss Rate	0.72**		0.76**																		
Amylose content	−0.93**	−0.68*	−0.94**	−0.71*																	
Leached materials																					
Starch Content																					
Amylose Content	−0.90**	−0.88**	−0.87**	−0.63*	0.86**																
Protein Content																					
Total solids	0.83**		0.85**	0.63*	−0.91**		−0.74**														
Starch weight	0.84**	0.86**	0.65*	−0.90**		−0.73**		0.99**													
Amylose weight	−0.86**	−0.77**	−0.85**	−0.66*	0.90**		0.93**		−0.70*	−0.68*											
Protein weight	0.84**		0.88**	0.72**	−0.92**		−0.71**		0.86**	0.85**	−0.75**										
Molecular parameters of Leached AP																					
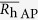		0.70*	0.78**	0.65*		−0.62*		−0.89**				−0.075**									
*X*_Ap1_																					
*X*_Ap2_														−0.64*	0.79**						
*h*_Ap2_		−0.79**					0.81**				0.66*		−0.85**		0.82**						
6 < *X* ≤ 12	0.66*	0.77**	0.61*		−0.65*		−0.83**				−0.65*		0.90**	−0.58*	−0.83**	−0.91**					
12 < *X* ≤ 24	0.76**	0.88**	0.71*		−0.69*		−0.94**				−0.84**		0.89**		−0.64*	−0.95**	0.89**				
24 < *X* ≤ 36	0.93**	0.78**	0.92**	0.67*	−0.90**		−0.91**		0.78**	0.78**	−0.88**	0.78**	0.76**				0.62*	0.76**			
36 < *X* ≤ 100	−0.78**	−0.88**	−0.73**		0.70*		0.95**		−0.59*	−0.58*	0.81**		−0.93**		0.69*	0.94**	−0.95**	−0.98**	−0.77**		
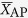																					

The content is the corresponding percentage (%) in the leached materials. The weight is the corresponding weight (mg per g rice kernel) in the leached materials, which is calculated by total solids of leached materials time the corresponding percentage. ^**^Correlation is significant at the 0.01 level (2-tailed); ^*^Correlation is significant at the 0.05 level (2-tailed).
